# Algal Active Ingredients and Their Involvement in Managing Diabetic Mellitus

**DOI:** 10.3390/biology13110904

**Published:** 2024-11-06

**Authors:** Aijun Tong, Dengwei Wang, Nan Jia, Ying Zheng, Yusong Qiu, Weichao Chen, Hesham R. El-Seed, Chao Zhao

**Affiliations:** 1 College of Tea and Food Science, Wuyi University, Wuyishan 354300, China; tajlove@163.com; 2 Department of Chronic and Noncommunicable Disease Control and Prevention, Fujian Provincial Center for Disease Control and Prevention, Fuzhou 350012, China; wdw2023@126.com; 3 State Key Laboratory of Mariculture Breeding, Key Laboratory of Marine Biotechnology of Fujian Province, Fujian Agriculture and Forestry University, Fuzhou 350002, Chinachenweichao@fafu.edu.cn (W.C.); 4 College of Marine Sciences, Fujian Agriculture and Forestry University, Fuzhou 350002, China; 5 College of Food Science, Fujian Agriculture and Forestry University, Fuzhou 350002, China; 6 Department of Chemistry, Faculty of Science, Islamic University of Madinah, Madinah 42351, Saudi Arabia; 7 International Research Center for Food Nutrition and Safety, Jiangsu University, Zhenjiang 212013, China

**Keywords:** algae, active substances, hyperglycemia

## Abstract

Diabetes mellitus (DM) is becoming increasingly prominent, posing a serious threat to human health. Its prevalence is rising every year, and often affects young people. In the past few decades, research on marine algae has been recognized as a major field of drug discovery. Seaweed active substances, including algal polysaccharides, algal polyphenols, algal unsaturated fatty acids, and algal dietary fiber, have unique biological activities. This article reviews the effects and mechanisms of the types, structures, and compositions of seaweed on inhibiting glucose and lipid metabolism disorders, with a focus on the inhibitory effect of active substances on blood glucose reduction. The aim is to provide a basis for the development of seaweed active substance hypoglycemic drugs.

## Simple Summary

Diabetes mellitus is becoming an increasingly serious health issue, and the bioactive compounds found in marine algae may offer promising options for its management. These compounds include polysaccharides, polyphenols, unsaturated fatty acids, and dietary fiber, all of which have the potential to disrupt glucose and lipid metabolism disorders. However, more research is needed to understand how these compounds work, determine the best dosages, and find effective delivery methods for managing diabetes. While substances derived from algae, such as fucoidan and red algal polysaccharides, show potential, further studies are required to identify specific beneficial compounds, establish safe consumption levels, and assess their long-term safety. Additionally, the low bioavailability of algae and the impact of their cell walls on nutrient absorption need to be addressed. Despite these challenges, adding algae to a balanced diet could serve as an alternative to traditional diabetes treatments, with the possibility of broader medical applications once their safety and effectiveness are validated.

## 1. Introduction

**Figure 1:**
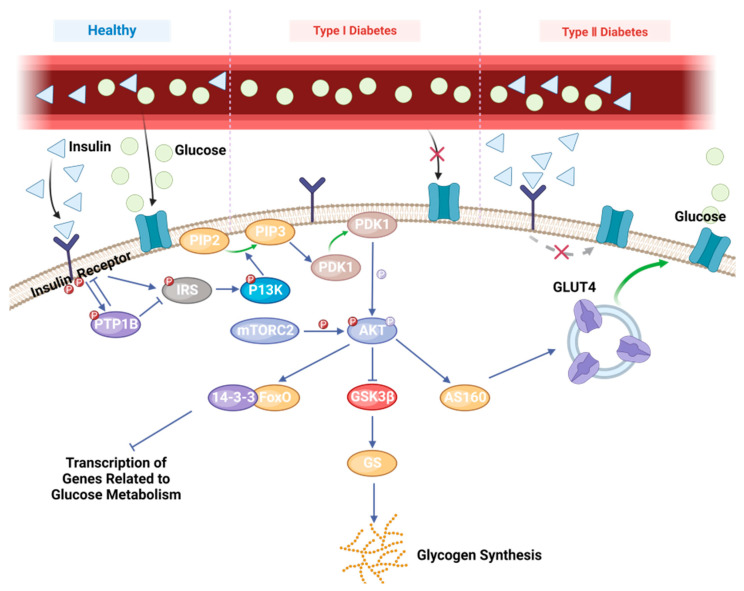
T1DM, T2DM and the AKT blood glucose regulation mechanism.

The most common endocrine and metabolic condition is diabetes mellitus (DM), which is brought on by either an absolute or insufficient release of insulin. The number of people with diabetes is increasing annually due to the aging population worldwide. Many people with diabetes experience other health problems that seriously compromise their quality and length of life. Type 1 diabetes mellitus (T1DM) and type 2 diabetes mellitus (T2DM) are the two main forms of DM [1]. Of these, T2DM (15 per 100,000 people) is thought to be more prevalent than T1DM (6059 cases per 1 billion people) [2,3]. According to a survey by the Diabetes Coalition, the global prevalence of diabetes in adults was as high as 10.5% in 2021, and this will increase to 12.2% by 2045 [4]. T1DM is a disease caused by autoimmune system-mediated islet cell hypoplasia or islet cell damage, leading to insulin deficiency. This condition is most common in the adolescent population and is usually treated by insulin injections [5]. T1DM is primarily caused by an autoimmune response that leads to the destruction of pancreatic beta cells, which results in an absolute insulin deficiency, while T2DM is the result of a combination of the body’s weakened response to insulin (insulin resistance) and a relative insulin deficiency (Figure 1).

T2DM is a chronic metabolic disease characterized by abnormally high blood glucose levels, which are primarily due to insufficient insulin production or a weakening of the body’s response to insulin, known as insulin resistance. During the development of T2DM, the beta cells in the pancreas usually gradually lose their ability to produce insulin, resulting in a drop in insulin production to about half of healthy levels [6]. In a healthy person’s body, pancreatic beta cells can precisely regulate the release of insulin to keep blood glucose levels stable. When people eat, glucose levels in the blood rise, and the pancreatic beta cells respond to this change by secreting the right amount of insulin. Insulin is a key hormone that helps the body’s cells absorb glucose from the blood and either convert it into energy or store it, thereby lowering blood sugar levels. However, in people with T2DM, this regulatory mechanism is disturbed. Due to reduced insulin secretion or the body’s reduced sensitivity to insulin, blood glucose levels are not effectively controlled, resulting in a persistent rise in blood glucose. Prolonged hyperglycemia can cause damage to blood vessels, nerves, kidneys, and other organs, increasing the risk of complications such as heart disease, stroke, kidney disease, and loss of vision [7]. Patients with postprandial hyperglycemia consume food with reduced insulin secretion and reduced inhibition of glucagon release, which leads to abnormal glucose levels in the liver and kidneys, reduced glucose uptake by cells, and increased blood glucose levels [8]. The rate at which ingested endogenous glucose enters the circulatory system exceeds the removal rate, which can lead to prolonged high levels of glucose, leading to loss of control of postprandial glucose homeostasis in the body and subsequent diabetes mellitus [9,10].

The insulin signaling pathway is essential for the prevention and management of diabetes, which regulates blood glucose levels by enhancing insulin sensitivity or facilitating insulin release to improve glucose metabolism in insulin-resistant states [11]. In this process, phosphatidylinositol-3-kinase (PI3K), insulin receptor substrate 1 (IRS1), and glucose transporter protein 4 (GLUT4) play central roles. When insulin binds to the insulin receptor on the cell surface, it activates tyrosine kinase activity within the receptor, a step that is critical for initiating insulin signaling (Figure 1) [12,13,14]. The activated insulin receptor in turn activates PI3K, an enzyme that plays a pivotal role in intracellular signaling. Activation of PI3K facilitates the translocation of the glucose transporter protein, GLUT4, from the intracellular to the cellular membrane, thereby increasing the uptake of glucose by the cell. At the same time, activation of PI3K inhibits the activity of c-Jun N-terminal kinase 1 (JNK1), an enzyme associated with cellular stress response and inflammation, and its overactivation may interfere with insulin signaling pathways, leading to insulin resistance. By inhibiting JNK1, the insulin signaling pathway was able to reduce insulin resistance and improve glucose metabolism [15]. In addition, IRS1, as a linker protein between insulin receptor and PI3K, is essential for insulin signaling. Activation of IRS1 promotes PI3K activation, which further enhances GLUT4 translocation and glucose uptake. Thus, by regulating the activity of these key molecules, blood glucose levels can be effectively controlled and insulin resistance can be reduced, which is particularly important for the treatment of T2DM. Future therapeutic strategies may target these molecules and develop new drugs to enhance the efficacy of the insulin signaling pathway to manage diabetes more effectively [16].

There is a wide variety of medications available for the treatment of T2DM, including, but not limited to, biguanides, sulfonylureas, alpha-glucosidase inhibitors, insulin sensitizers, glucagon-like peptide-1 (GLP-1) receptor agonists, and dipeptidyl peptidase-4 (DPP-4) inhibitors [17,18]. These drugs work through different mechanisms to lower blood glucose levels and improve insulin resistance. Disappointingly, the existing medications for diabetes treatment do not completely reverse the process of diabetes, and while they help to control blood glucose levels, they can sometimes bring about adverse side effects, such as gastrointestinal reactions, allergic reactions, and liver damage. These limit their application. Therefore, the search for new, natural, and safe hypoglycemic drugs or bioactive compounds has become particularly urgent. In recent years, scientific studies have found that nutrients in some foods have a positive effect on the prevention of T2DM. For example, diets rich in whole grains, fruits, vegetables, legumes, and nuts, as well as moderate alcohol consumption and low intake of refined grains, red or processed meats, and sugar-sweetened beverages, have been shown to reduce the risk of diabetes and improve glycemic control and blood lipid levels in people with diabetes [19].

Algae, a group of organisms widely distributed in nature, are not only an important part of the marine ecosystem, but also an indispensable health food in the human diet. They are a food but also have some medicinal value, and their products are therefore often regarded as substances of both medicinal and food origin. Algae appear on people’s tables in various forms, from nori and kelp to spirulina, and they not only enrich our diet but also bring benefits to our health. Scientific studies have demonstrated that algae are rich in nutrients, including proteins, vitamins, minerals, and unique bioactive compounds such as polysaccharides, dietary fiber, and antioxidants. Together, these components help maintain cardiovascular health and regulate blood lipid levels, thereby reducing the risk of cardiovascular disease and hyperlipidemia [20]. In recent years, macroalgae in the oceans have received a lot of attention in research due to their abundance of biologically active natural products, which have been recognized as a possible new source for the development of effective antidiabetic drugs. These algae contain a variety of compounds, such as polysaccharides, proteins, vitamins, and minerals, which have been shown to have potential benefits in regulating blood glucose and improving insulin sensitivity. They have diverse mechanisms of action, including promoting insulin secretion, improving insulin receptor sensitivity, reducing intestinal absorption of glucose, and enhancing antioxidant defenses, thereby potentially helping to slow the progression of diabetes and ameliorate metabolic abnormalities. It is estimated that there are about 9000 species of macroalgae in the world, which are classified into four main groups based on their pigment composition: red algae, brown algae, green algae, and cyanobacteria [21].

**Figure 2:**
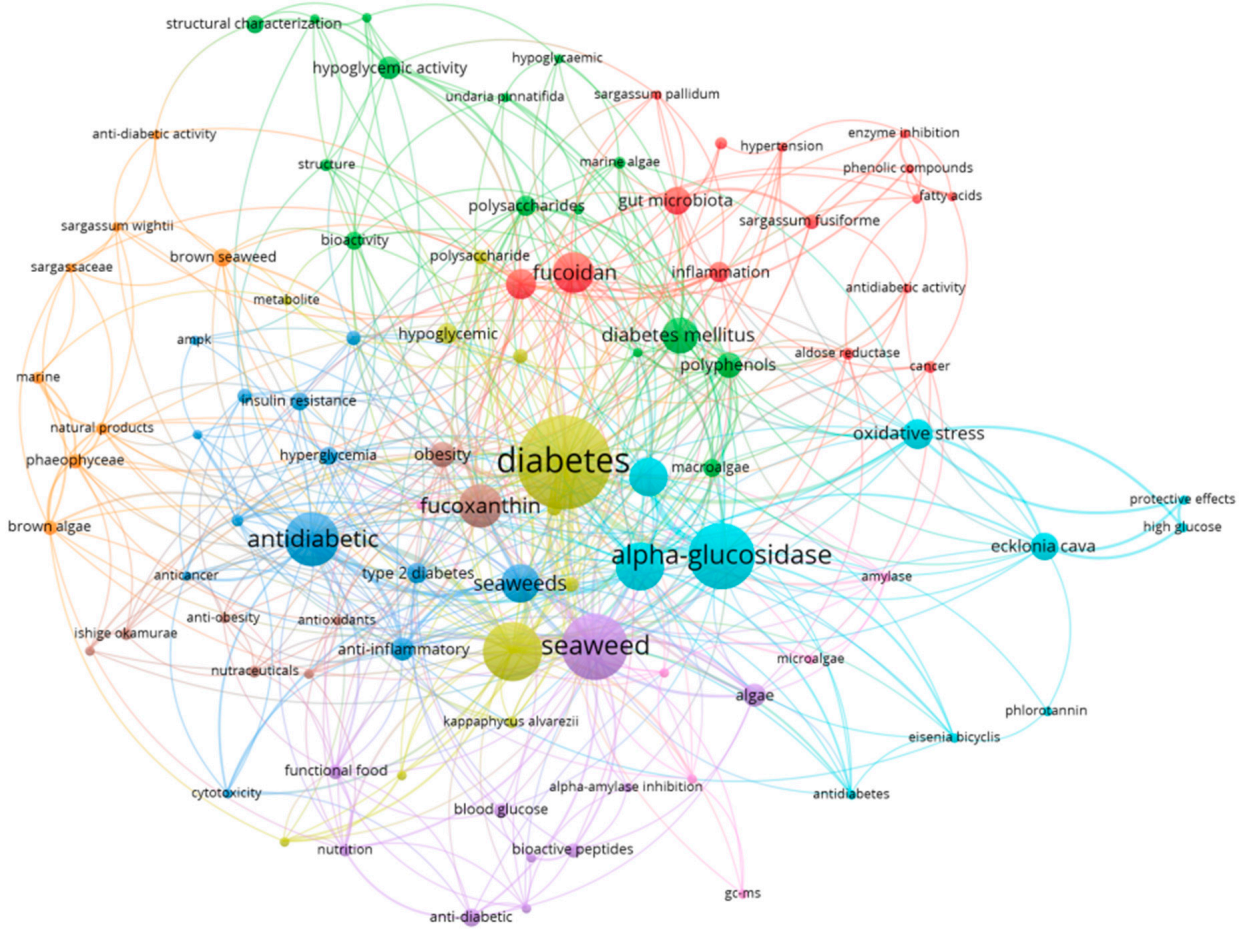
Visual network diagram of seaweed reducing blood sugar between 1989 and 2024.

Red algae are a class of algae living mainly in the ocean which occupies an important position in marine algae; examples include *Porphyra* and *Polysiphonia*. The application prospect of red algae extract in hypoglycemia is quite broad. Red algae polysaccharides can have certain potential for the prevention and treatment of diabetes by regulating lipid metabolism and lowering blood glucose levels [22]. The lectins in red algae extract can be used as functional food ingredients for diabetic people to help control blood glucose levels [23]. Brown algae are a group of algae that is widely distributed in the global ocean, and has a variety of economic and medicinal uses, such as lowering sugar and lipid levels, anti-inflammatory, antioxidant, and so on [24]. Brown algae contain a variety of components beneficial to human health, such as polysaccharides, polyphenols, fucoxanthin, and so on [25]. Green algae are rich in protein and many vitamins and minerals. Numerous studies have shown chlorella’s ability to enhance insulin responsiveness and reduce inflammation, with potential therapeutic benefits for T2DM [26,27]. Cyanobacteria, also known as blue-green algae, are bacteria capable of photosynthesis. For example, spirulina is rich in protein and vitamins and is used as a health food supplement [28]. In addition, some cyanobacteria can also produce bioactive substances, which have potential application value in drug development [29]. In recent years, seaweeds have received increasing attention, especially for their potential therapeutic role in hypoglycemic disorders, and a great deal of research has been conducted on their pharmacological effects, mechanisms of action, and clinical applications. However, the existing reviews on their pharmacological activities and mechanisms of action are still insufficient. Therefore, this review has screened and summarized the literature related to seaweed hypoglycemia with the intention of systematically reviewing the roles and mechanisms of seaweeds in inhibiting disorders of glucose metabolism by their types and compositions, with an emphasis on the inhibitory effects of the activities on blood glucose lowering. The aim is to provide a basis for the development of seaweed active substance hypoglycemic drugs. The co-occurrence networks of keywords in the field of seaweed reducing blood sugar from 1989 to 2024 are shown in this work (Figure 2). The high-frequency keywords were “seaweed”, “hypoglycemic”, and “diabetic”, indicating that during the last 35 years, in vitro research on bioactive compounds from algae with hypoglycemic properties has been a hotspot for research. CiteSpace was used to do a clustering analysis on all publications published in the field of hypoglycemic bioactive compounds from algae between 1989 and 2024 based on the co-occurrence of keywords.

## 2. Mechanisms of Hypoglycemic Activity

In T1DM, the patient’s pancreatic beta cells are attacked by an autoimmune response, resulting in the inability of these cells to produce insulin any longer, which triggers an absolute lack of insulin. This autoimmune damage is a progressive process that eventually leads to the patient’s need to rely on exogenous insulin to maintain normal blood glucose levels and life [30]. The human leukocyte antigen (HLA) gene region and the insulin gene region are the loci most strongly associated with the risk of developing T1DM. The HLA gene region is located on chromosome 6 and contains several genes that are strongly associated with the immune response. The insulin gene region, on the other hand, contains genes that encode insulin, a key hormone that regulates blood glucose levels. Polymorphisms in the insulin gene are also associated with the risk of developing T1DM, although their effect is relatively small [31,32]. In T2DM, defects in insulin signaling may be associated with β-cell resistance to insulin, which may be due to impaired function of the insulin receptor or other molecular abnormalities in the insulin signaling pathway. For example, dysfunction of insulin receptor substrate (IRS) proteins or overactivation of phosphatases may lead to attenuated insulin signaling, which may affect the insulin secretion capacity of β-cells [8]. Recent scientific advances have revealed strong associations between T2DM and multiple genetic loci, and these findings provide new directions for the development of gene-based therapeutic strategies. For example, the insulin gene, the insulin receptor gene, the peroxisome proliferator-activated receptor (PPAR) gene, the glucokinase gene, and the hepatocyte nuclear factor-1alpha (HNF-1alpha) transcription factor gene have all been identified as key genetic factors influencing the risk of T2DM. By gaining a deeper understanding of how these genes affect insulin secretion, insulin signaling pathways, and glucose metabolism, researchers hope to find new ways to regulate the expression or function of these genes to reduce or treat the risk of T2DM [33,34,35,36,37]. *Ulva* polysaccharide (ULP) was able to regulate the expression of InsR and AMPK, as well as inhibit the expression of JNK, JAK, STAT3, p16, and p38, and improve the dysfunction of glucose metabolism [38]. Insulin-resistant HepG2 cells treated with fucoxanthin self-assembled nanoparticles showed a significant decrease in ROS formation, which was explained by the activation of the nuclear factor E2-related factor 2/heme oxygenase-1 (Nrf2/HO-1) pathway. Insulin receptor substrate 1/glucose transporter type 4 (IRS1/GLUT4) was subsequently activated, facilitating the uptake and use of glucose inside cells [39]. During the pathology of T2DM, the insulin secretory function of pancreatic β-cells is affected by glucose stimulation, and the production of reactive oxygen species (ROS) during this process may lead to oxidative stress, which in turn affects the health of β-cells. Therefore, when considering increasing insulin secretion using gene therapy to reduce the risk of diabetes, it is important to carefully assess the potential damage that such interventions may cause to β-cells [40].

The treatment of T2DM is based on a combination of strategies such as glucose lowering, blood pressure lowering, lipid regulation, anticoagulation, weight control, and lifestyle improvement. Glucose lowering is the ultimate treatment goal, and lifestyle improvement is the basic treatment measure, which should be carried out throughout the treatment. Once lifestyle fails to achieve glucose control and lowering, drug therapy needs to be started. The rapid breakdown of starch into glucose causes blood glucose levels to rise, a phenomenon that is a classic symptom of T2DM and is known as a postprandial blood glucose spike [41]. Dietary starch is broken down into large amounts of maltose by the enzyme alpha-amylase, which alpha-D-glucosidase then continues to break down into glucose. Therefore, by inhibiting the activity of alpha-amylase and alpha-D-glucosidase, blood glucose levels can be regulated [42,43]. Some studies have shown that fucoidan can inhibit the activity of α-glucosidase and α-amylase, delay the body’s intake of glucose, and reduce postprandial blood glucose levels [44]. The dual inhibitory effect of alpha-amylase and alpha-D-glucosidase helps to reduce the symptoms of hyperglycemia in diabetic patients. Meanwhile, the development of type 2 diabetes is driven by two key factors: reduced insulin sensitivity and failure of pancreatic beta cell function [45,46].

**Figure 3:**
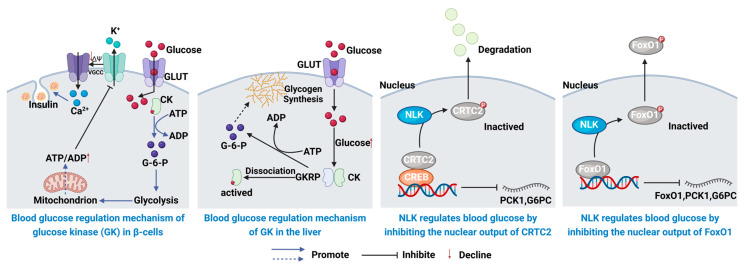
Glucose metabolism pathway-based targets and therapeutic medicines [48].

Hexokinase IV (GK) is a key enzyme in glucose metabolism, catalyzing the phosphorylation of glucose to glucose-6-phosphate. Primarily expressed in pancreatic β-cells and hepatocytes, it also occurs in various other cells such as neurons and intestinal endocrine cells. Unlike hexokinases I–III, GK has a higher Km value and is not inhibited by glucose-6-phosphate. Structurally, GK comprises large, small, and linker domains, with the latter housing the glucose-binding site. GK operates in three conformations: closed, open, and super-open, reflecting different substrate states. In β-cells, GK facilitates glucose-induced insulin secretion (GSIS) by promoting ATP production, which regulates KATP channels and Ca^2+^ influx. In hepatocytes, GK is regulated by GKRP, forming an inactive complex under hypoglycemia, which dissociates as glucose levels rise to promote glycogen synthesis. NLK, a conserved pro-directed Ser/Thr kinase, initially identified in *Drosophila*, has been implicated in various cancers but more recently has been shown to regulate hepatic gluconeogenesis. NLK inhibits two key enzymes in gluconeogenesis, PCK1 and G6PC, via phosphorylation of CRTC2 and FoxO1. NLK-mediated CRTC2 degradation and FoxO1 nuclear export suppress gluconeogenesis. Reduced NLK expression in T2DM models suggests its role in disease progression, positioning NLK activators as potential therapeutic targets for T2DM (Figure 3). For example, polyphenolic compounds extracted from the brown alga *Ecklonia cava* can reduce fasting blood glucose and insulin levels, and this effect is associated with reduced hepatic gluconeogenesis due to decreased activity of glucose-6-phosphate dehydrogenase and inhibition of hepatocytes, and its mechanism of action is related to the overexpression of glucose-regulated genes such as phosphoenolpyruvate carboxykinase in T2DM [47]. Meanwhile, active substances such as polysaccharides and polyphenols in seaweeds may play a role in regulating glycolipid metabolism and lowering blood glucose levels by affecting the activity of key transcription factors such as FoxO1.

## 3. Algal Active Substances

**Figure 4:**
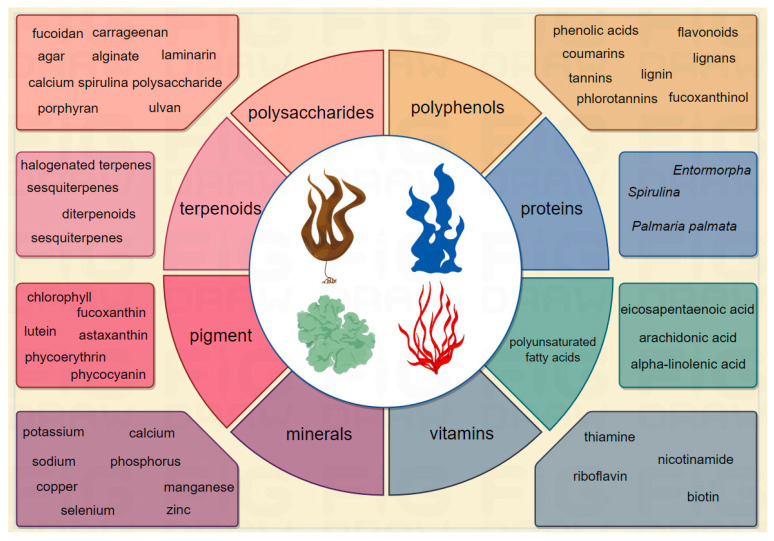
Algal bioactive substances.

The active substances of seaweeds mainly include polysaccharides, polyphenols, proteins, fatty acids, vitamins, minerals, pigment, terpenoids, and other active substances [49]. The active substances of seaweed are shown in Figure 4, among them, seaweed polysaccharides including fucoidan, carrageenan, agar, alginate, laminarin, calcium, spirulina, polysaccharide, and so on. Seaweed polyphenols include phenolic acids, flavonoids, coumarins, tannins, lignin, lignans, phlorotannins, and so on. Seaweed active substances have the advantages of being natural, new, and unique, and are receiving more and more widespread attention [50]. These active substances exist in the extracellular matrix, cell wall, protoplasm, and primary and secondary metabolites in the cell, which not only play an important role in the physiological and biochemical processes of seaweeds, but also have high economic value and potential for pharmaceutical development. These active substances have been shown to have anti-inflammatory, antioxidant, and hypoglycemic effects, and are capable of affecting many pathways related to insulin production, glucose metabolism, and insulin sensitivity, providing a comprehensive approach to the management of diabetes and contributing to the development of marine algae as antidiabetic drugs [51,52,53]. According to studies, carrageenan in seaweed polysaccharides may compete with FITC-insulin to bind to the insulin receptor, disrupting PI3K and AKT activation and so reducing the production of glycogen synthase and glucose transporters [54]. Fucoxanthin influences the development of insulin resistance and T2DM by attenuating low-grade inflammation in adipocytes [55]. Traditional antidiabetic drugs, such as metformin and sulfonylureas, focus primarily on enhancing insulin sensitivity and stimulating insulin secretion. However, these drugs are often accompanied by side effects and limitations in long-term efficacy. In contrast, seaweeds have multiple advantages as antidiabetic agents, including their abundance of biologically active components, multiple mechanisms for regulating blood glucose, and their availability and affordability as a natural resource. Compared with chemically synthesized drugs, they have fewer side effects and are more suitable for long-term consumption or as a supplement to medication.

### 3.1. Hypoglycemic Effect of Algal Polysaccharides

Polysaccharides are the main components of algae, accounting for approximately 20% to 70% of the dry weight [56]. These algal polysaccharides exhibit various biological activities, such as antiradiation, antitumor, antioxidation, and antiviral effects [57,58,59]. As shown in Table 1, the polysaccharide components found in plants do not raise blood glucose levels; rather, they have the ability to lower them. These polysaccharides work by inhibiting gluconeogenesis, a process that generates glucose in the body, through the regulation of key enzymes involved in glucose metabolism. Additionally, they promote the synthesis of glycogen in the liver, which is crucial for maintaining stable blood sugar levels. Furthermore, these polysaccharides can enhance insulin secretion due to their hypoglycemic effects, helping to correct abnormalities in glucose metabolism and combat insulin resistance [60]. Research in recent years has shown that algal polysaccharides can also inhibit the activity of amylase and sucrase, and retard blood glucose. Studies have shown that polysaccharides and oligosaccharides from seaweed could reduce hyperglycemia, promote insulin secretion, improve glucose tolerance, lower blood glucose and urine glucose, and alleviate other symptoms in diabetic mice induced by alloxan [61]. Algal polysaccharides are considered to be potential candidates for blood glucose regulation due to their unique structural features such as degree of sulfation, sugar chain structure, molecular weight, type of glycosidic bond, and degree of branching [62].

#### 3.1.1. Alginate Polysaccharides

##### *Sargassum fusiforme* Polysaccharide

The combination of *S. fusiforme* polysaccharide (SFP)—an acidic, water-soluble polysaccharide extracted from *S. fusiforme*—with low-dose acarbose significantly improved diabetic symptoms and serum markers, demonstrating a better antidiabetic effect than acarbose alone. Particularly, the combination therapy of SFP with low-dose acarbose was able to activate the IRS/PI3K/AKT signaling pathway in terms of regulating fasting blood glucose, improving insulin resistance, and reducing renal damage. In addition, the combination therapy helped prevent hepatic fat accumulation by regulating the expression of HMGCR and SREBP-1c genes. SFP also showed positive effects in restoring damaged renal tissues and improving renal function, significantly reducing the expression of blood urea nitrogen (BUN), B-cell receptor (BCr), 24 h urine protein, TGF-β1, and Smad7 mRNA. This suggests that SFP may delay and prevent diabetes-induced kidney injury by inhibiting the activation of the TGF-β1/Smad signaling pathway. These findings provide new strategies and therapeutic ideas for the prevention and treatment of diabetes and its complications [63]. When SFPS I interacted with α-glucosidase, the semi-inhibitory concentration (IC50) of SFPS I was 0.31 mg/mL when the concentration of SFPS I reached 0.55 mg/mL, which was able to completely inhibit the activity of α-glucosidase. This inhibition was reversible, and SFPS I inhibited the enzyme activity by decreasing its catalytic rate rather than causing a permanent loss of enzyme activity. The binding of SFPS I to α-glucosidase caused a significant change in the tertiary structure of the latter. SFPS I first binds to α-glucosidase to form an enzyme-inhibitor complex (EI), and subsequently, when the substrate pNPG is added, an enzyme–inhibitor–substrate (EIS) complex is formed. The EIS complex reduces or inhibits enzyme catalysis [64]. It has been found that the alleviation of diabetes symptoms may be closely related to the regulatory effect of Sargassum fucoidan (SFF) on the intestinal flora. In an animal model of hyperglycemia induced by streptozotocin (STZ), SFF was effective in reducing the fasting blood glucose level of mice, as well as their food intake and water intake. In addition, SFF had the effect of attenuating cardiac and hepatic pathological changes, improving liver function, and inhibiting the occurrence of oxidative stress. Meanwhile, SFF also significantly modulated the composition of gut microorganisms in the feces of diabetic mice, reducing the relative number of diabetes-associated intestinal bacteria, which is thought to be one of the possible mechanisms by which SFF alleviates the symptoms of diabetes [65]. The hypoglycemic effects of the polysaccharides in different studies showed some consistency, but there were some variations in their activity due to the extraction method, purity, the growth environment of the plant, and the chemical composition and structural properties of the polysaccharides. Different extraction and purification techniques may lead to differences in the chemical composition and structural properties of the polysaccharides of *S. fusiforme*, thus affecting their hypoglycemic activity. For example, sheepshead polysaccharide (SFP) prepared by the enzymatic digestion ultrasound-assisted hot water method showed a higher extraction rate and α-glucosidase inhibitory activity. The chemical composition of sheepshead polysaccharides, such as the content of glucuronic acid and sulfate groups, as well as the composition and order of arrangement of the monosaccharides, had a significant effect on their hypoglycemic activity. Two different molecular weight polysaccharide fractions, SFP-1 and SFP-2, were obtained by ultrafiltration. SFP-2 has the higher total sugar and glucuronic acid content, while SFP-1 has the higher sulfate group content, and these differences may lead to their differences in hypoglycemic activity [66].

##### *Laminaria japonica* Polysaccharide

*L. japonica* polysaccharide (LJP) is one of the main functional factors in *L. japonica*, a bioactive substance with many physiological functions related to its bioactive polysaccharides. Currently, fucoidan is one of the main components of LJP. Researchers have found that LJP has a significant positive effect on T2DM in mice. LJP exhibits the potential to lower blood glucose by modulating serum levels of insulin and glucagon. Low molecular weight fucoidan with a high degree of sulfation has stronger antidiabetic effects, while derivatives of persulfated fucoidan have far stronger hypoglycemic effects. Furthermore, the only alternatively linked fucoidans that had a greater inhibitory impact on α-D-glucosidase were (1,3) and (1,4) [67,68]. Notably, LJP intake not only prevented weight loss in diabetic mice, but also effectively lowered fasting blood glucose (FBG), total cholesterol (TC), triglycerides (TG), and low-density lipoprotein cholesterol (LDL-C) levels, while elevating serum insulin and high-density lipoprotein cholesterol (HDL-C) levels. These effects suggest that LJP may be a promising candidate for the development of natural drugs for the treatment of diabetes [69,70]. Studies have shown that LJP has a significant hypoglycemic effect, possibly through stimulation of insulin release from the pancreas or reduction in metabolic rate. In addition, LJP improved lipid levels, suggesting its potential in diabetes management [71].

#### 3.1.2. Green Algal Polysaccharides

##### *Ulva lactuca* Polysaccharide

*U. lactuca*, a genus of Ulva and a phylum of green algae, is a group of green algae that is widely distributed worldwide and often causes widespread green tides due to overgrowth, which has attracted the attention of many countries. The green alga *U. lactuca* possesses many potential bioactive substances and functional properties, especially polysaccharides. ULP is an intercellular product, which exists in the form of heteropolysaccharide in algae, containing monosaccharides such as D-glucose, D-xylose, L-rhamnose and D-glucuronide. Compared with algae products such as fucoidan, fucoidan agar, agar gum, and carrageenan, research on ULP started later. ULP has anti-inflammatory, antioxidant, antiviral, antitumor, lipid metabolism regulation, and blood sugar regulation effects [72]. Numerous previous studies have confirmed the ability of ULP to alleviate the symptoms of type 2 diabetes by reducing blood glucose levels through enhanced insulin sensitivity, as well as boosting superoxide dismutase (SOD) and catalase (CAT) activity [73,74]. ULP can also play a positive role in regulating blood glucose levels by activating the antioxidant defense system, such as enhancing the activity of glutathione peroxidase (GPx), effectively resisting the attack of excess free radicals, protecting cells from oxidative damage, inhibiting the formation of hazardous substances in the process of lipid peroxidation, and maintaining the integrity and function of the cell membrane [75]. Ruan et al. administered *U. lactuca* water extract and ULP to normal mice and alloxan-induced diabetes mice, respectively. The results showed that *U. lactuca* water extract and ULP had a hypoglycemic effect on alloxan-induced diabetes mice, but had no effect on normal mice, and ULP had a better hypoglycemic effect than *U. lactuca* water extract [38].

##### *Enteromorpha prolifera* Polysaccharide

Potential synergistic effects between *E. prolifera* polysaccharide (EPP) and other antidiabetic compounds or therapeutic approaches are mainly characterized by the ability to inhibit α-glucosidase activity, thereby slowing down the digestion and absorption of carbohydrates, and improving glycemic control through the activation of the IRS1/PI3K/AKT pathway and mediation of the intestinal flora, enhancing the action of insulin. EPP has been found to have a significant positive effect on T2DM in a mouse model. These polysaccharides showed the potential to lower blood glucose by modulating serum levels of insulin and glucagon. The study also found that EPP intake not only prevented weight loss in diabetic mice, but was also effective in lowering FBG, TC, TG, and LDL-C levels, while boosting serum insulin and HDL-C levels. These results suggest that EPP may exert its hypoglycemic effect by enhancing the expression of InsR protein in the liver and pancreas. In addition, EPP significantly altered the intestinal microbiota in the feces of diabetic mice, reducing the relative abundance of intestinal bacteria associated with diabetes, which may be one of the potential mechanisms by which EPP suppresses diabetic symptoms [76]. The author has reported that FBG levels and oral glucose tolerance test (OGTT) results decreased significantly in diabetic rats, implying that EPP ameliorated glucose metabolism in T2DM rats. Histopathological evidence showed that EPP reduced oxidative damage stress in tissue, thereby preventing diabetic complications in the liver, pancreas, and jejunum tissues [77]. Yuan et al. evaluated the antioxidant activity and antidiabetic effect of EPP: firstly, the degradation of EPP produced marshmallow oligosaccharides, whose average molecular weight was determined by gel permeation chromatography analysis to be 44.1 kDa; the main monosaccharide composition of marshmallow polysaccharides was determined by capillary electrophoresis to be rhamnose, glucuronic acid, glucose, xylose, and galactose. In vitro studies have indicated that oligosaccharides from *E. prolifera* exhibit strong reducing and antioxidant effects, such as scavenging DPPH, superoxide, and nitric oxide radicals. In vivo studies have shown that *E. prolifera* oligosaccharides could reduce excessive drinking, eating, wasting, and hyperglycemia in high-glucose and high-fat adjuvant streptozotocin (STZ)-induced diabetic mice [78]. It was demonstrated that *E. prolifera* oligosaccharides could promote insulin secretion by reducing pancreatic inflammation and apoptosis in diabetic mice. Lin et al. studied the effect of EPP on glucose metabolism in diabetic rats and found that EPP could improve glucose metabolism; the mechanism may be related to the antioxidant activity of EPP and its ability to regulate the mRNA levels of InsR, GCK, APN, and GLUT-4 genes in liver and adipose tissue, indicating the potential ability of EPP in the treatment of diabetes [79].

#### 3.1.3. Others

*Porphyra*, which belongs to the red algae, *Reduviidae*, is an important cultured red algae along the coast of China. *Porphyra* is rich in nutrients, such as protein, carbohydrates, fat, vitamins, and so on. In addition to that it is also rich in active ingredients, such as *Porphyra* polysaccharide, *Porphyra* protein, active peptides, etc. The polysaccharide extracted from *Porphyra* is an acidic polysaccharide that contains a high number of sulfate groups. Numerous studies have confirmed that this polysaccharide is not only an important bioactive substance but also has significant health benefits, including anti-aging, antitumor, anticoagulation, and ulcer prevention. In particular, some studies have pointed out that polysaccharides in nori can play a positive role in maintaining human health through a variety of mechanisms, such as regulating the intestinal microbial community, enhancing immune function, providing antioxidant protection, and exhibiting antidiabetic, antimicrobial, and lipid-lowering effects. In addition, by inhibiting α-amylase activity, *Porphyra* polysaccharides show potential for lowering blood glucose, especially in a diabetic rat model. It may become a natural drug resource for the treatment of T2DM in the future and has the potential to be developed as an ingredient in nutraceuticals and functional foods [80].

At present, there are few studies on cyanobacterial polysaccharides, mainly represented by *Spirulina*, which is considered to be a heteropolysaccharide composed of various monosaccharides. It was shown that the combination of aerobic exercise and *Spirulina* polysaccharide (SP) supplementation significantly enhanced learning and memory ability in T2DM rats, and this improvement may be related to better regulation of the expression of proteins related to the p75NTR signaling pathway, which may inhibit apoptosis in the hippocampal region of T2DM rats [81]. *Spirulina* supplementation has beneficial effects on controlling blood glucose levels and improving lipid profile in T2DM patients [82].

**Table 1:** Hypoglycemic effects of algal polysaccharides.

Algal Sources	Effects	References
*Sargassum fusiforme*	Preventing the TGF-β1/Smad signaling pathway from being activated	[63,83]
	After joining forces with α-glucosidase to generate an enzyme-inhibitor (EI) complex, EIS compounds will bind to the substrate pNPG to create their compounds. EIS substances reduce or prevent an enzyme’s capacity to catalyze reactions.	[64]
	Modifies the gut microbiota in diabetic mice’s excrement and lowers the proportion of intestinal bacteria linked to diabetes	[65]
*Laminaria japonica*	The regulation of insulin and amylin in serum	[69]
	Increases serum insulin and HDL-C levels	[70]
	Enhances InsR protein expression in the liver and pancreas	[71]
*Ulva lactuca*	Enhances insulin sensitivity, raising SOD and CAT levels, thereby reducing blood sugar	[73,74]
	In vivo enhancement of antioxidant activity and reduction in lipid peroxidation damage	[75]
*Enteromorpha prolifera*	Reduces oxidative damage stress in tissue, thereby preventing diabetic complications in the liver, pancreas, and jejunum tissues	[77]
	Reduces inflammation and apoptosis to stimulate insulin secretion	[78]
	Regulates the mRNA levels of InsR, GCK, APN, and GLUT-4 genes in the liver and adipose tissue	[79]
*Porphyra*	Inhibits α-amylase	[80]
*Spirulina*	Increases p75NTR signal-related protein expression in rats with T2DM, inhibiting apoptosis	[81]

### 3.2. Hypoglycemic Effect of Algal Polyphenols

Polyphenols in marine algae show the potential to reduce the likelihood of certain diseases. Both seaweed polyphenols and terrestrial plant polyphenols have antidiabetic potential, but their mechanisms of action may differ. The antidiabetic effects of algal polyphenols may be more related to their antioxidant capacity and inhibition of digestive enzyme activity. These polyphenolic compounds, such as fucoidan polyphenols, flavonoids, phenolic acids, and halogenated phenols extracted from various species of seaweeds, have a wide range of biological properties that are beneficial to the human body. They not only possess powerful antioxidant properties, but also have anticancer, antimicrobial, anti-inflammatory, and antidiabetic activities. The antioxidant properties of these polyphenolic compounds are particularly prominent in their ability to neutralize free radicals and reduce oxidative stress, thereby protecting cells from damage. In addition, these compounds can regulate the body’s metabolic pathways, reduce inflammatory responses, inhibit the growth of cancer cells, and maintain the body’s glycometabolic balance by regulating blood sugar levels. Therefore, marine algal polyphenols have important applications in functional food and drug development, and they may be a natural resource for the prevention and treatment of many diseases [47,84,85]. Studies have shown that dichlorotannins extracted from marine macroalgae genera such as *Alaria*, *Pulmaria*, *Ecklonia*, and *Ascophyllum* have significant antidiabetic properties. These natural compounds effectively lower blood glucose by regulating insulin and glucagon levels in the body and have a preventive effect on weight loss in diabetic mice [86,87,88]. In addition, phlorotannins extracted from *Ecklonia cava* (*E. cava*) have shown positive effects on postprandial glycemic control in the obese population, i.e., they were able to significantly reduce the postprandial area under the glucose curve (AUC), lower fasting blood glucose levels, reduce fasting plasma insulin concentrations, improve the homeostasis model assessment of insulin resistance (HOMA-) IR) indices, and effectively reduce glycated hemoglobin (HbA1c) levels [89,90].

#### 3.2.1. Phlorotannins

Phlorotannins are the signature constituents of algae and consist of only one basic unit, mesotriol (1,3,5-trihydroxybenzene). These unique compounds form a series of linear and reticulated polymers of varying molecular weights that are present in algae such as *Ecklonia*, *Eisenia*, and *Ishige*. Numerous studies have demonstrated that phlorotannins possess a variety of biological activities, including antidiabetic, anticancer, and antibacterial effects [91,92]. Polyphenols extracted from the brown alga *Ecklonia stolonifera* had α-glucosidase inhibitory activity and hypoglycemic effect [93]. Polyphenolic compounds isolated from *Sargassum hemiphyllum* showed inhibitory activity against α-amylase, α-glucosidase, sucrase, and maltase, as well as the ability to promote insulin secretion [94]. Phenolic compounds extracted from *Ascophyllum nodosum* exhibit strong inhibitory activity against α-glucosidase as well as have the potential to influence glycemic control in vivo [95,96,97]. Phlorotannins are extracted by a combination of ultrasound-assisted extraction, microwave extraction, and enzyme-assisted extraction [98]. The isolation and purification of phlorotannins for potential pharmaceutical applications face challenges in terms of extraction efficiency, removal of impurities, the complexity of alginate polyphenol chemical structure, stability, economics, and bioavailability.

#### 3.2.2. Red Algal Polyphenols

Two bromophenols purified from the red alga *Grateloupia elliptica*, 2,4,6-tribromophenol and 2,4-bromophenol, have specific chemical structures. 2,4,6-tribromophenol, with the chemical formula C_6_H_3_Br_3_O, is a white or off-white lamellar crystal having a relative density of 2.55, a melting point of 95–96 °C, and a boiling point of about 244 °C. It is soluble in water, but can be dissolved in ethanol, ether, isopropyl alcohol, acetone, methyl ethyl ketone, chloroform, and toluene. It is almost insoluble in water, but can be dissolved in ethanol, ether, isopropanol, acetone, methyl ethyl ketone, chloroform, toluene, and other organic solvents; the chemical formula of 2,4-bromophenol is C_6_H_4_Br_2_O and is usually a white or light yellow solid to liquid. These bromophenol compounds not only have diverse biological activities, including antioxidant, anticancer, antibacterial, and antidiabetic, due to their unique chemical structures, but also have potential applications in the pharmaceutical and food industries [99]. Bis-(2,3-dibromo-4,5-dihydroxyphenyl)-methane 7 was reported as a natural bromophenol with a significant inhibitory effect on the negative regulator protein tyrosine phosphatase 1B (PTP1B) isolated from the red alga *Rhodomela confertavies* [100]. Five bromophenol compounds isolated from the red alga, including 3′,5′,6′,6-tetrabromo-2,4-dimethyldiphenyl ether, 1,2,5-tribromo-3-bromoamino-7-bromomethylnaphthalene, 2,5,8-tribromo-3-bromoamino-7-bromomethylnaphthalene, 2,5,6-tribromo-3-bromoamino-7-bromomethylnaphthalene, and 2′,5′,6′,5,6-pentabromo-3′,4′,3,4-*tetramethoxybenzo*-phenone, all showed significant inhibition of protein tyrosine phosphatase 1B (PTP1B). The chemical structures of these compounds are highly brominated, which may be related to their biological activities. Therefore, these bromophenol compounds derived from red algae may have promising applications in the field of diabetes treatment [101].

### 3.3. Hypoglycemic Effect of Algal Unsaturated Fatty Acids

Seaweeds are an important class of marine living resources that contain polyunsaturated fatty acids (PUFAs), especially omega-3 and omega-6 fatty acids, which are essential for human health. These fatty acids play a variety of physiological roles in the human body, including maintaining cell membrane fluidity, regulating inflammatory responses, and promoting brain development. Omega-3 fatty acids, such as eicosapentaenoic acid (EPA) and docosahexaenoic acid (DHA), have been shown to have significant benefits in terms of lowering the risk of cardiovascular disease, improving brain function, and have anti-inflammatory properties. The amount of these polyunsaturated fatty acids in seaweed is about 2% of its dry weight, which is not a significant amount, but its high PUFA fraction and low omega-6:omega-3 ratio make seaweed a powerful supplement to other sources [102,103]. Lipids are essential nutrients for the survival and growth of all organisms, and are important structural components of cell membranes, which play an important role in the energy storage of many organisms [104]. Algae are a treasure trove of unsaturated fatty acids, notably monounsaturated fatty acids (MUFAs) and polyunsaturated fatty acids (PUFA), which have been widely recognized for their health-promoting properties [105]. Because of the growing problems of overfishing and marine pollution, algal sources of PUFAs offer a more environmentally friendly and sustainable alternative to extracting PUFAs from deep-sea fish. These nutrients are particularly noteworthy for their potential role in mitigating the risk of T2DM, a prevalent lifestyle-related disease. Among these, microalgae stand out as a rich and promising source of essential metabolites, including omega-3 long-chain PUFAs, which play a crucial role in maintaining human health and could be instrumental in the management and prevention of T2DM [106]. The study found that a high carbohydrate diet would not improve the insulin sensitivity of non-diabetes subjects, because monounsaturated fatty acids and polyunsaturated fatty acids could reduce the level of low-density lipoprotein cholesterol, but would not have adverse effects [107]. The effects of monounsaturated fatty acids (MUFAs) and saturated fatty acids (SFAs) on insulin action and glucose utilization in rat L6 skeletal muscle cells have been compared, and the results indicate that SFAs and MUFAs have a significant effect on insulin signaling and glucose uptake in L6 muscle cells and suggest that a MUFA-rich diet may contribute to normal and insulin-resistant skeletal muscle glucose uptake and utilization [108]. Formula NBF1, which is rich in seaweed fiber and n-3 PUFA, was found to positively affect C-reactive protein (CRP) and reduce adiponectin (APN) and diabetes-related markers. Specifically, intake of seaweed fiber and n-3 PUFA was positively associated with increased lipocalin levels, while intake of n-3 PUFA was inversely associated with decreased CRP levels [109].

### 3.4. Hypoglycemic Effect of Algal Dietary Fiber

As the seventh major nutrient, dietary fiber has attracted more and more attention for its excellent performance in improving advocacy function and lowering blood fat, cholesterol, etc. The rich dietary fiber resources in algae are cheaper than other dietary fiber resources. The relatively simple composition of algae also makes dietary fiber easier to extract and utilize. Moreover, the microalgae dietary fiber rich in the sulfonic acid group has more abundant physiological functions and has better prospects in the production of functional food. In recent decades, scholars at home and abroad have conducted a lot of research on the preparation and function of algae dietary fiber, and have made rich research achievements. A large intake of dietary fiber can significantly reduce the occurrence of T2DM [110]. Consumption of dietary fiber, especially soluble fiber, above the amount recommended by the American Diabetes Association (ADA) significantly improves glycemic management in people with T2DM, reduces high insulin levels, and helps to lower blood lipid levels [111]. It has been shown that taking seaweed dietary fiber can effectively reduce fasting and postprandial blood glucose, as well as significantly reduce triglyceride levels and increase HDL levels [112].

### 3.5. Hypoglycemic Effect of Algal Peptides

Algal peptides exhibit a variety of bioactive characteristics due to their unique amino acid composition and structure. Studies have shown that algal peptides possess biological activities such as antioxidant, antibacterial, anti-inflammatory and antitumor [113,114]. Algal peptides are gaining attention as a novel bioactive substance, particularly with the deeper understanding of metabolic diseases. DPP-4 is an important enzyme involved in the regulation of insulin secretion and glucose metabolism; therefore, its inhibitors are of great significance in the treatment of metabolic diseases such as diabetes mellitus. Studies have shown that algal peptides exert inhibitory effects on DPP-4 through multiple mechanisms, which in turn affect blood glucose levels and insulin sensitivity [115]. Hydrolyzed peptides of seaweed proteins inhibit the activity of α-glucosidase and α-amylase in the digestive tract, thereby reducing blood glucose levels and sugar absorption. These peptides also inhibit the activity of DPP-4, which increases the signaling of the insulin-like growth factor-I receptor and promotes insulin secretion, as well as the proliferation and differentiation of small intestinal cells. In addition, hydrolyzed peptides of seaweed proteins can increase the level of GLP-1, thereby enhancing satiety and reducing energy intake [116,117,118]. The interaction of seaweed peptides with DPP-4 was studied using molecular docking analysis, which revealed the structural basis and mechanism of action by which seaweed peptides inhibit DPP-4. In the study, it was found that the complex of seaweed peptides with DPP-4 was stabilized by CH-π interactions, specifically with key amino acid residues of DPP-4, thereby inhibiting its activity [119].

## 4. Conclusions

In summary, marine macroalgae are a viable resource for the development of natural antidiabetic medicines due to their vast variety of bioactive chemicals. Algal compounds such as fucoidan and red algal polysaccharides (agar-oligosaccharides) are the most promising for use in diabetes management, but key issues such as structural characterization of these compounds, conformational relationships and mechanisms of action, effective dosages, and optimal delivery modes are not yet clear, and these need to be further advanced by improving analytical techniques and conducting clinical analyses to achieve targeted and efficient application of bioactive ingredients. For example, the bioactivity of seaweed peptides is maintained during the extraction process, while avoiding the destruction of other sensitive active ingredients, thus improving the overall extraction efficiency. However, more study is needed to pinpoint the precise chemicals causing these effects, figure out the right quantities, and comprehend the long-term safety and effectiveness of consuming macroalgae in managing diabetes. When the human body directly ingests seaweed, its bioavailability is low, and the indigestible seaweed cell walls may also impede the absorption of the active ingredients in the intestinal tract. When combined with other lifestyle changes and a balanced diet, including macroalgae may provide an alternative to traditional diabetic therapies.

Addressing potential side effects associated with consuming algae active ingredients involves several aspects. First, a focus on exploring algae germplasm that possesses high nutritional value, special biological activity, or excellent production performance. Second, the development of new and high-value processing technologies for algal food to enhance the added value of algal products. Third, the establishment and improvement of the safety evaluation system for algal food to clarify the hazard factors affecting algal safety and their mechanisms of action, such as evaluating their toxicity and side effects through in vitro and in vivo experiments to ensure their safety in clinical applications. The future application of seaweed active compounds in the medical field holds rich and unlimited possibilities. In terms of drug development, it is anticipated that effective drugs can be synthesized based on these active substances to combat challenging diseases. Meanwhile, the pharmacokinetic properties of seaweed active substances, including their stability and bioavailability in vivo, still need to be further investigated, and in order to address these issues the development of effective delivery systems, such as nanoparticles or liposomes, to improve their stability and bioavailability. Furthermore, the exploration of additional pharmacological activities and potential applications requires the efforts of the scientific community.
